# Cefazolin access and use in Ethiopia: A policy implication

**DOI:** 10.1371/journal.pgph.0001421

**Published:** 2023-01-17

**Authors:** Getachew Alemkere, Asres Teshome, Gobezie Temesgen, Getnet Abebe, Yidnekachew Degefaw, Hiwot Tilahun, Workineh Getahun, Eshetu Girma, Wondwossen Amogne

**Affiliations:** 1 Department of Pharmacology and Clinical Pharmacy, School of Pharmacy, College of Health Sciences, Addis Ababa University, Addis Ababa, Ethiopia; 2 Department of Surgery, School of Medicine, College of Health Sciences, Addis Ababa University, Addis Ababa, Ethiopia; 3 Antimicrobial Resistance Prevention and Control Case Team, Pharmaceuticals and Medical Equipment Directorate, Ministry of Health, Addis Ababa, Ethiopia; 4 Department of Surgery, Gimjabet Primary Hospital, Gimjabet, Amhara, Ethiopia; 5 Antimicrobial Resistance and Global Partnership, USAID Medicines, Technologies, and Pharmaceutical Services Program, Management Sciences for Health, Addis Ababa, Ethiopia; 6 Department of Preventive Medicine, School of Public Health, College of Health Sciences, Addis Ababa University, Addis Ababa, Ethiopia; 7 Department of Infectious Diseases, School of Medicine, College of Health Sciences, Addis Ababa University, Addis Ababa, Ethiopia; Swiss Federal Institute of Technology Zurich: Eidgenossische Technische Hochschule Zurich, SWITZERLAND

## Abstract

Healthcare systems in resource-limited nations have been challenged by the shortage of essential medicines. This study explores cefazolin access and uses history in the Ethiopian healthcare delivery system, for possible policy implications. An exploratory qualitative study was conducted from July to August 2021. Semi-structured questions and observation guides were used to extract necessary data from people, documents, and field visits to hospitals, government supply agencies, and pharmaceutical business firms. The data were transcribed, coded, organized into themes, and presented. Cefazoline is the recommended first-line surgical antibiotic prophylaxis (SAP) in the Ethiopian Standard Treatment Guideline (STG) and is included in the national Essential Medicine List (EML). However, it was not available for use in the Ethiopian pharmaceutical markets for years. While the shortage might stem from supply-demand mismatches, multiple unknown issues exist in the background of the shortage. This is evidenced by the removal of cefazolin from the recent government procurement list regardless of the recommendation set in the national EML and STG. This study found a historic shortage of cefazolin in Ethiopian healthcare settings. This implies that the antibiotic availability in the pull market may not reflect required usage at facilities for several reasons including the misalignment of national guidelines and national procurement processes, and miscommunication between pharmacies and clinicians at sites on drug availability. Changing the essential medicines list and/or procurement requests without active review of the supply chain system and prescribing practices at facilities can lead to the elimination of necessary antimicrobial agents from the national public health sector supply.

## Introduction

The healthcare systems have been challenged by the shortage of essential medicines [1–3]. As summarized by Shafiq et al., many essential antibiotics (including cefazolin) are in short supply at different times across different countries [4]. This antibiotic shortage has posed a global health threat [4, 5]. The shortage is associated with a lack of access to effective therapies and associated increased mortality. In addition, resorting to an alternative treatment in response to the unavailability of the preferred agent may have public health and economic implications [2, 6].

Cefazolin shortage has been reported in Japan [3] and India [7]. These shortages have confused the drug choice and resulted in increased use of third-generation cephalosporins like ceftriaxone and cefotaxime, from the watch category according to WHO AWaRe classification [5, 8, 9]. Japan’s studies had also indicated a poor treatment outcome and increased cost due to the use of such alternative agents [3, 5, 10].

There is inadequate data regarding cefazolin access and its associated effects in Ethiopia. Cefazolin is indicated as a first-line agent for surgical antibiotic prophylaxis (SAP) in the recent national standard treatment guideline (STG) for general hospitals [11]. However, dozens of studies on SAP showed a high rate of inappropriate use for SAP on one hand due to inappropriate drug choice [12–18]. In all these studies, ceftriaxone alone or in combination with metronidazole are the major drugs used in place despite the guideline recommending cefazolin. Unlike the experiences in Japan, the effect of such alternative broad-spectrum antimicrobial use is not studied in Ethiopia [5, 8, 10]. However, other studies had shown a high burden of surgical site infections in Ethiopian hospitals despite extensive prescribing of prophylactic agents [19, 20]. These were attributed to the guideline non-concordant use secondary to shortage of first-line antibiotics like cefazolin, intended to cover gram-positive cocci introduced during skin incision [13]. Studies had demonstrated the efficacy of cefazolin as prophylaxis over alternative antimicrobial in lowering surgical site infections [21].

The use of cefazolin is not also limited to surgery. The above-mentioned recent Ethiopian STG indicated cefazolin for musculoskeletal infections, pneumonia (as an alternative), and skin-soft tissue infections [11]. In support of these recommendations, evidence was showing the superiority of cefazolin over alternative antibiotics (like ceftriaxone) for the management of Methicillin Sensitive Staphylococcus Aureus (MSSA) bacteremia [22–24].

The current research, therefore, aims to assess cefazolin access and use history in the Ethiopian healthcare delivery system including a desk review of cefazolin inclusion in the guidelines, medicine lists, pharmaceutical supply, and procurement lists as well as field visits for the pharmaceutical stores and collecting practitioners’ experience towards cefazolin.

## Methods and materials

### Study setting and contexts

**Fig 1:**
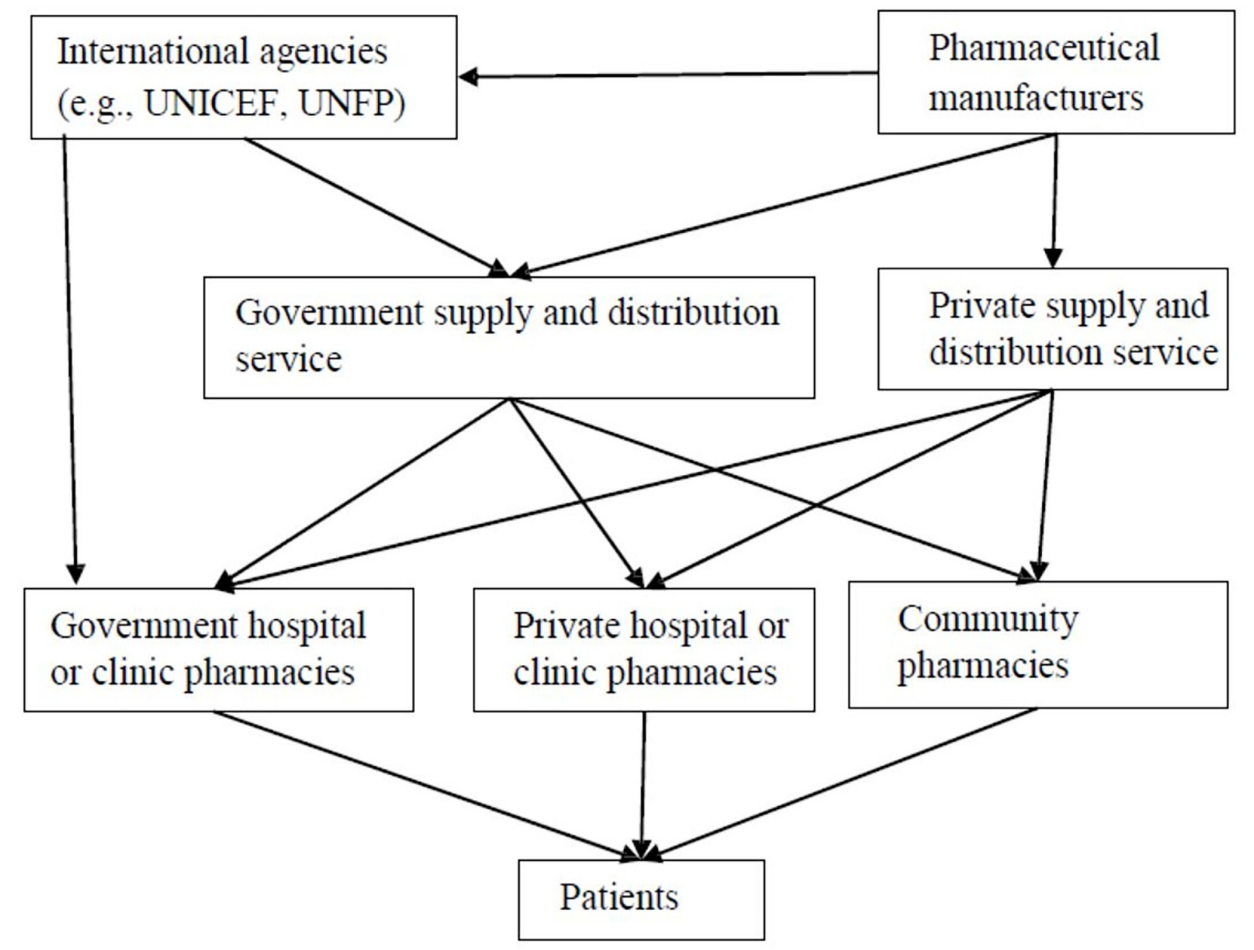
The Ethiopian Pharmaceutical supply and distribution system. Abbreviations: UNICEF: United Nations Children’s Fund, UNFP: United Nations Population Fund.

The Ethiopian Pharmaceutical Supply Agency (EPSA) is one of the single largest government-owned drug distributors and importers. The agency procures drugs from international agencies, local manufacturers, and importers. It also involves in direct medicine import. It is primarily responsible for the distribution of medicines for government health facilities and rarely for private healthcare facilities. The pharmaceutical supply system is largely affected by the controlling mechanisms of the Ethiopia Food and Drug Authority (EFDA) and the Ethiopian Customs Commission (ECC). Its function is also influenced by the national guidelines and EMLs as well as facility epidemiologic data, facility medicine lists, and other guiding documents and prescribing practices (Fig 1).

The study was conducted in the EPSA and three hospitals in Addis Ababa, namely Tikur Anbessa Specialized Hospital (TASH), Dagmawi Menelik Hospital (DMH), and Zewditu Memorial Hospital (ZMH). The hospitals were selected conveniently as they receive the largest volume of surgical referral cases that are expected to use cefazolin prescriptions. TASH is the only top largest referral and teaching hospital in Ethiopia. The hospital has 800 beds, of which 299 beds are dedicated to surgery. DMH and ZMH are comprehensive specialized hospitals. DMH serves as a teaching hospital and a specialty center for ophthalmology services. ZMH is also a teaching center and delivers several medical and surgical care. These hospitals have outpatient, in-patients, and emergency pharmacies. Besides, ten pharmaceutical business firms attending the Ethiopian Pharmaceutical Association (EPA) annual conference had been visited for their product lists available for promotion.

### Study design and period

A qualitative exploratory study design with a purposive sampling technique was conducted using key informant interviews and observation guides. The study was conducted between July to August 2021.

### Study participants and recruitment process

Pharmacists working in the selected hospitals and the EPSA were part of the study population. Particularly, pharmacy heads, the supply management unit and store heads of each selected hospital, and other key personnel indicated by the staff were included. A purposive sampling technique was used to choose the study participants. All pharmaceutical firms involved in antimicrobial manufacturing, import, retail, and distribution, that participated in the EPA annual conference, were also targeted.

### Data collection process

The data were generated from targeted desk reviews, field visits with an observational checklist, and key informant interviews.

#### Targeted desk review

This was the first step in the data collection process. National resources, particularly the treatment guidelines, essential medicine lists, EPSA procurement lists, and other relevant documents were reviewed. All the freely available STGs, EMLs, and procumbent lists in the Ethiopian Ministry of Health, Ethiopian Food and Medicine Authority (EFDA), EPSA website, and other pages were reviewed, without restrictions.

#### Filed visits and document review

Memos were used to visit the EPSA warehouse, stores of three selected hospitals and its associated documentation, as well as product lists of ten available pharmaceutical firms regarding cefazolin coverage. For the pharmaceutical business firms, only the product lists available during the conference were visited and the sales representatives were briefly interviewed. Moreover, any recent pharmaceutical procurement/supply agreement documents and facility medicine lists, as well as antimicrobial leftover/expiry reports in the past three years were reviewed for the hospitals and EPSA.

#### Key informant interviews

Interviews were made with nine selected study participants who were willing to take part in the study. The key informant interview was prepared based on the findings of the desk review. The interview questionnaire has three sections including the sociodemographics, the cefazolin supply (cefazolin inclusion in the procurement lists, history of supply, and leftover/expired stock), and its use (uses based on the STG and EML, a consequence of such deviations from STGs, the role to mitigate the case, readiness to address the gap if conditions fulfilled) sections. The cefazolin supply and use section is restructured differently for the EPSA and hospital interviewees to fit it to their specific context of practice.

The data was collected by two of the authors (GA and AT). While AT was involved in the interview administration, hospital visits, and document reviews, GA was involved in the desk review, the business firm visit, and interviews. The data collection guide was drafted by GA and AT and then validated by all the research teams. The research methodology and data collection training were delivered by the qualitative research expert who is part of this research (EG). In addition, consecutive modifications were made and reviewed once the desk review was completed.

### Data analysis

The data collected was transcribed, skimmed, coded, categorized/organized into themes manually, and presented textually. AT transcribed and coded the data. Then, GA recoded and thematized the data iteratively.

### Ethical approval

The ethical review committee of Addis Ababa University, School of Pharmacy approved the study. Written consent was taken, and an interview and document review were then conducted. The anonymity of respondents was ensured in each data collection and analysis process.

## Result

### Desk review

The recent STGs, EMLs, and pharmaceutical procurement lists (PPL) were reviewed. The three recent STG editions recommend cefazolin as a first-line agent for SAP [11, 25]. The recent STG also recommends cefazolin for osteomyelitis, pneumonia, and skin infections (e.g., cellulitis) [11]. Besides, cefazolin is included in the latest editions of the EMLs [26–28]. In contrast, once used to be included in the sole government supplier, EPSA procurement list, it has been removed since 2020/2021 [29, 30]. Hence, it was not currently available and used in Ethiopian healthcare delivery systems (Table 1).

**Table 1:** Cefazolin inclusion in the national guidelines and medicine lists, July to August 2021.

Document type and year of publication	Cefazolin covered	Use/indication/	Reference
STG 4^th^ edition, 2021	Yes	Surgical prophylaxis: musculoskeletal infections, pneumonia (add on), Skin soft tissue infections	[11]
STG 3^rd^ edition, 2014	Yes	Surgical prophylaxis	[25]
STG 2^nd^ edition, 2010	Yes	Surgical prophylaxis	
EML 6^th^ edition, 2020	Yes	Listed under antibacterial for access antibiotics and powder for injection form	[28]
EML 5^th^ edition, 2015	Yes	Listed under antibacterial powder for injection form	[27]
EML 4^th^ edition, 2002	No		[26]
PPL 2^nd^ edition, 2020	No		[30]
PPL 1^st^ edition, 2018	Yes	Read as ‘Cepz-32: Cephazoline Sodium - 1gm—Injection with Diluent’	[29]

Abbreviations: STG-standard treatment guideline, EML-essential medicine list, PPL-pharmaceutical procurement list

### Field visits and document review

The field visit showed that cefazolin was not available in the stores of any of the visited hospitals and EPSA. All studied facilities have medicine lists and procurement agreements with EPSA. Cefazolin was covered in the unrevised medicine list and older procurement agreements.

Also, none of the ten pharmaceutical business firms available for promotion at the EPA annual conference had included cefazolin in their product lists. The managers or medical representatives of those promoting firms also mentioned as they did not have encounters with cefazolin manufacturing, import, and sales. They noted that the absence of the market demand for cefazolin and the poor awareness about cefazolin’s place of therapy might have contributed to the occurrence.

### Key informant interviews

#### Characteristics of study participants

Nine key individuals from EPSA and selected hospitals were interviewed, four of them were from EPSA. All of them were pharmacists and males. Three participants were from TASH, one is the pharmacy head, and the remaining two were drug supply management (DSM) and drug information center (DIC) heads. The remaining two participants were pharmacy heads from DMH and ZMH. The majority of them were bachelor’s degree holders. All have at least seven years of pharmacy experience and at least two years of position-specific experience. Only two pharmacists working at hospitals had received training within the last two years. EPSA’s responders had worked in forecasting and quantification leadership positions, pharmaceutical supply, and warehouse head positions (Table 2).

**Table 2:** General information of study participants, July to August 2021.

Participant institution	Age	Education level	Experience (years)	Experience, Position (year)	Related training within 2 years	Participant code
(IA1)	34	B.pharm	10	10	No	A01
(IA2)	38	B.pharm	12	12	No	A02
(IA3)	41	B.pharm	15	11	No	A03
(IA4)	44	B.pharm	16	8	No	A04
(IB1)	31	B.pharm	7	3	Yes	B05
(IB2)	39	BSC; MBA	10	2	Yes	B06
(IB3)	42	MSC	13	2	No	B07
(IC1)	43	MSC	20	-	No	C08
(ID1)	40	B.pharm	14	8	No	D09

Abbreviations: B.Pharm; Bachelor of Pharmacy. MSc: Masters, MBA: Master of Business Administration, MD: medical doctor, IA/IB/IC/ID: Institution A/B/C/D

EPSA interviewees suggested different reasons for the omission of cefazolin in the recent PPL edition [30]. Most of them mentioned a declining cefazolin demand from the facilities. For the question ’How EPSA decide on the drugs to be included in the EML and the reasons behind how this mismatch was created,’ one of the responders said ‘demand and an expert suggestion’ were the frequently used parameters to add or remove medicines from the list. The respondents acknowledged the opportunities of reconsidering its inclusion in the next revision if the demand is created and if the suggestion come from the practitioners.

We also tried to find out how the procurement agreement has been made between them (i.e., EPSA and the hospitals). EPSA regularly updates the facilities about the recently available drugs to be requested and supplied. Procuring the missed essential medications from the non-EPSA suppliers are left to the hospitals. Unfortunately, the government facilities visited have negligible procurement agreement experiences with the private suppliers and this depends on the management’s willingness and budget availability. Accordingly, a supply head working in a hospital replied that the agreement was determined majorly on the bases of the budget allocated for pharmaceuticals by the hospital. The cost, consumption amount, and the essentiality of the drug will also be considered to redistribute the allocated budget for the purchase.

*“…………*..*Even though the budget allocation majorly depends on the consumption rate and requirement and essentiality of the drug; the drug cost and availability also matter during budget allocation*. *If the drug is cheap and easily available in any other pharmacy*, *giving priority to those unavailable drugs and the costly drug is usually mandatory since the price to afford this medication is much better here than private pharmacies due to margin of profit difference*.*”* (a 39-year-old hospital pharmacist, 10 years of experience)

#### Cefazolin procurement and supply history

All the participants from the hospitals mentioned that they only have a procurement agreement with EPSA. And all of them did not remember a cefazolin procurement agreement with EPSA. However, one participant disclosed other means of receiving the drug. He said *“…one to two years back we got Cefazolin from ‘MSF-Spain’…*… *In different times we get different drugs from other foreign countries by aid… especially when a shortage of drugs encounters*.*”* (a 39-year-old hospital pharmacist, 10 years of experience)

The EPSA participants mentioned that they had been procuring cefazolin previously, ‘around 3 years back’. The procurement decreased from time to time and completely ceased recently. The possible reason suggested by the EPSA participant regarding the decline in cefazolin procurement and its ultimate deletion from the PPL includes source problems, PPL expert suggestion, poor demand, and clinician preference for alternative drugs.

*“Yes*, *we have been supplying cefazolin previously*, *around three years back*. *However*, *we did not handle it currently*, *due to declining demand from the healthcare facilities*, *source problems*, *and high wastage rates probably due to preferences to recent generation cephalosporins”* (a 34-age pharmacist, 10 years experience)

#### Cefazolin leftover/expired stock history

Most of the participants in EPSA and hospitals responded as they did not encounter cefazolin leftover/expired stock, recently. However, a hospital pharmacy head mentioned there was a history of unused and ultimately expired cefazolin stock. However, he did not comment on the wastage amount and other related details. The reason for expiration, as he remembers was related to ‘miscommunications between the store and outpatient pharmacy with the in-patient pharmacies.’

*“Despite it being in our medicine list*, *for the last three years*, *we did not receive cefazolin from EPSA*. *However*, *we were able to receive it from a donor*, *before around three years*, *but ultimately expired due to poor communications among the store and dispensary pharmacy units*.*”* (a 39-year-old hospital pharmacist, 10 years of experience)

This might also be related to the poor demand for the drug by the prescribers. It should be seen as a missed communication point between the pharmacy department and the prescriber teams. Otherwise, except few recent records, we did not access older records to verify the details of the occurrence.

#### Participants’ awareness and their readiness to change the practice

Most of the respondents affirm that the drug choice for surgical prophylaxis and skin source of infection management depends on the anticipated etiology. The respondents believe that ceftriaxone use, over cefazolin, may have consequences including increased cost and side effects like a high risk of resistance induction.

We also attempted to see their willingness to change the practice based on the guideline recommendations. All the participants were willing to change their current practice once cefazolin is made available and awareness is created among all the practitioners. Participants from EPSA said ’there is an amendment period for the PPL. Hence, there is room to reconsider it once the demand is created or the practicing physicians suggest doing so.’ (a 34-age pharmacist, 10 years of experience, and a 38-age pharmacist, 12 years of experience)

## Discussion

The research aims to assess cefazolin access and use in the Ethiopian healthcare delivery system. Specifically, aimed to review cefazolin presence in the national documents, health facilities records, pharmaceutical stores, and collect practitioners’ experience on cefazolin access and use in Ethiopia.

Cefazolin was not available in the Ethiopian healthcare delivery system despite its inclusion in the recent national STGs and EMLs due to various bottlenecks in its supply and use. In place, alternative drugs were used for cases that can be managed by cefazolin such as for surgical prophylaxis. While the shortage might stem from supply-demand mismatches, multiple unknown issues exist in the background of the shortage.

Globally, many essential antibiotics are in short supply [1–4]. The context in LMICs may have different contexts [2]. Action to address the shortage of antimicrobials should be at the forefront especially to promote antimicrobial drug management [4]. However, countries and institutions are trapped in the urgency dilemma to settle it. A comprehensive plan and international cooperation are needed to secure the continuous supply and availability of essential antibiotics.

Some of the challenges in the Ethiopian context were the exclusion of essential antibiotics from the procurement lists of the government and private suppliers. Cefazolin was included in the previous PPL [29] of the major government supplier, EPSA, but omitted from the current edition [30]. EPSA regularly updates the available drugs for the facilities. Procuring the missed essential medications from the non-EPSA suppliers are left to the hospitals. Unfortunately, the government facilities visited have negligible procurement agreement experiences with the private suppliers.

Inequities in healthcare evidence dissemination, the absence of policy backups, and poor attention to the problems from the management might be contributing factors to the cefazolin shortage in the national market pool. In the absence of a clear demand or a policy that supports it, other suppliers or importers are primarily money-driven, so they might not import an antibiotic with a specific purpose. Rather, they focus on importing or supplying broader spectrum antibiotics with multiple uses. The global antibiotic shortage experiences might have also contributed [1–4].

In Ethiopia, due to the above problems and other unrecognized bottlenecks, there is a widespread culture of prescribing broad-spectrum antibiotics for surgical prophylaxis, such as ceftriaxone only, and in combination with other antibiotics [12, 13, 19, 31]. This widespread alternative drug use (probably driven by a ‘broader better’ mantra or poor communication on the available antibiotics) might be the havoc that had been pressuring poor cefazolin supply that continued to distort the supply-demand pursuit behavior of users. This is an expensive decision for at least two reasons. One, most surgical procedures bypass the skin whereby cefazolin is more effective to cover the skin floras than ceftriaxone [21]. Second, ceftriaxone is a broad-spectrum antibiotic with a higher potential of inducing selective pressure for antimicrobial resistance than cefazolin [32]. That is why it is classified as a watch group by the World Health Organization [9] and Ethiopian EML [28]. More impeding to this decision is that the changes experienced in the healthcare delivery system may go to the extent of deleting such first-line access antibiotics from the pharmaceutical procurement lists and institutional medicine lists [30]. Besides, the TASH pharmacy department had reported the occurrence of expired cefazolin stocks due to poor communication.

Such practices can bring a shift in the consumer behavior towards the use of limited broad-spectrum antibiotics, suck hospital budget, introduce AMR and force utilization of the next level of defenses (e.g., demand for carbapenem use secondary to high rate of extended-spectrum beta-lactamase-producing bacteria associated with ceftriaxone use) [32]. Generally, these problems may increase healthcare costs and hampers antimicrobial stewardship program initiatives across the country [32].

The current study provides important evidence that helps to build a case for the provision and supply of the needed antibiotic within the Ethiopian health system. However, it had engaged limited but some key stakeholders in the supply and use system. The engagement of more clinicians and experts from the drug administration authority might have added some critical insights. In addition, except for narrating the occurrence, it did not investigate the depth of the background reasons for the shortage across all stakeholders. Furthermore, although the study explored the supply history back a few years, it did not follow a historic study methodology and did not present trends of supply and use changes.

## Conclusion

Demand for specific antibiotics in the market pull systems may not reflect required usage at facilities for several reasons including the misalignment of national guidelines and national procurement processes, alternative private and donor procurement at the sub-national level, and miscommunication between pharmacies and clinicians at sites on drug availability. Changing the essential medicines list and/or procurement requests without active review of the supply chain system and prescribing practices at facilities can lead to the elimination of necessary antimicrobial agents from the national public health sector supply. Enhanced communication across the service delivery lines and a policy might be required to ensure access to cefazolin or suggest alternatives in place so that antimicrobial stewardship programs function properly.
